# Extramedullary Solitary Plasmacytoma with Anaplastic Features of the Nasopharynx

**DOI:** 10.1155/2020/8845546

**Published:** 2020-07-23

**Authors:** Parikshit Padhi, Radwa El-Behery

**Affiliations:** ^1^Memorial Medical Cancer Center, 2530 S Telshor Blvd, Las Cruces, NM 88011, USA; ^2^Integrated Oncology, 5005 S 40th St, Phoenix, AZ 85040, USA

## Abstract

Extramedullary plasmacytomas (ESPs) are rare forms of plasma cell dyscrasias and usually are seen in the upper aerodigestive tract. ESPs with anaplastic features are extremely rare, and no treatment guidelines exist. We present a 75-year-old gentleman presented with left nasal blockage, and on examination, a polypoid left nasal mass was seen. He was, then, referred to ENT after a CT scan revealed a mass in the left nasopharynx for a biopsy. The preliminary reports suggested a high-grade lymphoma; however, after further testing, it was revealed to be an anaplastic plasma cell neoplasm. PET scan, bone marrow biopsy, serum and urine protein electropheresis, serum immunofixation and light chains were all unremarkable for systemic disease. Differential diagnosis included plasmablastic lymphoma, NK/T cell lymphoma-nasal type, and squamous cell cancers of the head and neck. He was treated with radiation alone given his comorbidities. Given there are no treatment guidelines, we would like to highlight this rare case and discuss different potential management options such as radiation, chemotherapy, surgery, or a combination of different modalities.

## 1. Introduction

Extramedullary plasmacytomas (ESP) are rare forms of plasma cell dyscrasias with anaplastic variants mentioned in only a few case reports. There are no guidelines for the management of ESP; however, most are treated with radiation alone since they tend to be radiosensitive. It is unclear whether ESP with anaplastic features require more aggressive management with radiation and chemotherapy. This entity can resemble plasmablastic lymphoma, and it is paramount to rule out plasmablastic lymphoma since the management is different. We present a case of ESP with anaplastic features of the nasopharynx and will discuss the features and management of this rare entity.

## 2. Case Presentation

A 75-year-old gentleman with a history of Usher Syndrome with retinitis pigmentosa presented to his family doctor because of a sensation of left-sided nasal blockage especially on deep inspiration. He denied any nasal pain, nasal swelling, or epistaxis. He was legally blind in both eyes but had minimal vision in his left eye. On examination of his nose, a pink-colored polypoid mass was arising from the left nasal orifice. The extension could not be determined on physical examination. He has no history of hepatitis, HIV, or any chronic viral illnesses. He was a former smoker with a 60 pack year history, but no IV drug use or alcohol use. He had no family history of cancer.

**Figure 1:**
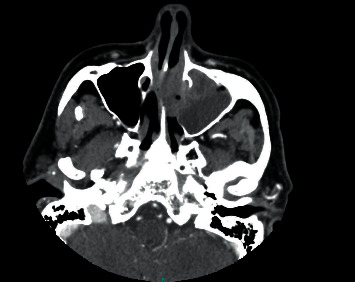
CT scan of the sinus showing an expansile mass in the left nasopharyngeal region leading to rightward nasal septum deviation and destruction of the etmoid cells.

**Figure 2:**
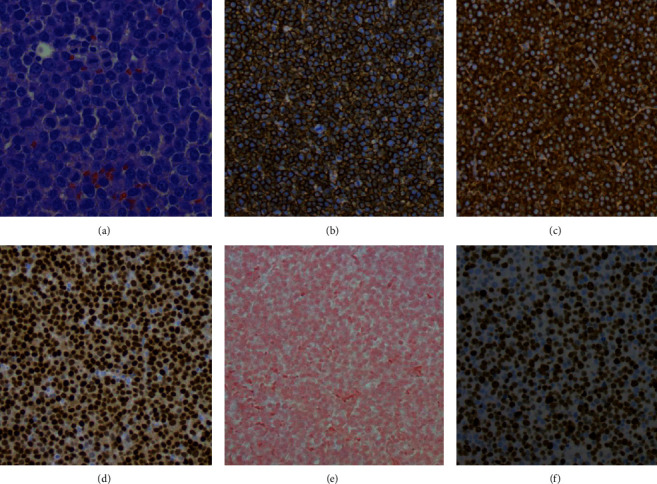
Nasopharyngeal mass biopsy reveals diffuse infiltrate of large atypical cells with variably eccentric neuclei, ampophilic cytoplasm, prominent nucleoli, and occasional uninucleated forms (a), that are CD138 positive (b), kappa restricted (c), MUM-1 positive (d), EBER negative (e), and a Ki-67 overall of 60% (f).

A computed tomography (CT) scan of the sinuses revealed a 3.1 × 4.4 × 3.2 cm mass arising from the left nasopharynx, causing rightward deviation of the nasal septum (Figure 1). He followed up with the ENT surgeon who performed a biopsy of this mass. Initially the frozen section revealed small round blue cells suggestive of an aggressive lymphoma, but it was sent for a second opinion. A CT scan of the chest, abdomen, and pelvis and, eventually, a PET scan showed no other sites of disease. Eventually, the biopsy from the nasopharyngeal mass revealed a diffuse infiltrate of large atypical cells with variably eccentric nuclei, fairly abundant amphophilic cytoplasm, prominent nucleoli, and occasional binucleated forms. Few Dutcher bodies were seen. Mitosis was markedly increased, and atypical mitotic figures were identified. Apoptosis was also increased, and focal necrosis was present. Immunostains revealed the neoplastic cells to be positive for CD38 (strong), CD138, CD79a, MUM-1, kappa, IgA, BCL-2, and CD43 and negative for CD20, PAX-5, CD45, CD30, EMA, EBER by ISH, ALK-1, HHV8, lambda, IgG, IgM, IgD, CD56, CD5, CD10, BCL-6, BCL-1, CAM5.2/AE1, and CD117. Ki-67 was positive, variable 30–80% and overall 60% (Figure 2). He was diagnosed with an anaplastic plasma cell neoplasm. He, then, came to our cancer center for further management. Initial blood tests included complete blood counts (white blood count: 6.22 × 10^3^/microlitre, hemoglobin: 15.9gm/dL, platelet count: 244 × 10^3^/microlitre, and absolute neutrophil count: 3.42 × 10^3^/microlitre), chemistries (sodium: 134 mmol/L, potassium: 4.3 mmol/L, chloride: 105 mmol/L, BUN: 17 mg/dL, creatinine: 0.86 mg/dL, and calcium: 10.2 mg/dL, and liver enzymes were within the normal range), and myeloma parameters (M protein: not observed, total protein: 7.2 gm/dL, albumin: 4 gm/dL, globulin 2.9 gm/gL, IgG 852 mg/dL, IgA 241 mg/dL, IgM 98 mg/dL, kappa light chains: 18.1 mg/L, lambda light chains: 13.7 mg/L, and kappa/lambda ratio: 1.34). A bone marrow biopsy revealed a normal cellular marrow for age (20–30%) with normal trilineage hematopoiesis, and no increased plasma cells were noted.

Our differentials included plasmablastic lymphoma, NK/T cell lymphoma, or a nasopharyngeal squamous cell carcinoma. We had to ensure that the patient did not have plasmablastic lymphoma as there is some overlap with the morphological and immunohistochemistry features with anaplastic plasma cell neoplasm. However, the absence of HIV history, the negative EBER by ISH, the presence of several Dutcher bodies, occasional binucleated forms, the Ki-67 that is well below 90%, the positive BCL-2, and negative EMA and CD30 are all in favor of a plasma cell neoplasm over a plasmablastic lymphoma. Histology analysis showed no evidence of a carcinoma or a, NK/T cell pathology.

We initially discussed with the patient that options include radiation to the nasophayngeal mass followed by either lenalidomide- or bortezomib-based therapy versus radiation alone. His case was discussed in our multidisciplinary tumor board, and based on his age, performance status, and comorbidities, it was decided to treat him with radiation alone (50 Gy/25 fractions) and reserve systemic treatment in the event of recurrence. He completed radiation treatment and, however, received 40 Gy due to the concern of complete blindness in his left eye. Postradiation PET scan and CT scan showed a complete response. Myeloma workup continued to show no evidence of systemic disease. He has remained in remission for 6 months, and we are monitoring him with imaging every 4–6 months and blood work (complete blood counts, chemistries, and myeloma parameters) every 3 months.

## 3. Discussion

Solitary plasmacytoma (SP) is a rare form of plasma cell dyscrasias in which neoplastic proliferation of monoclonal plasma cells are present in one location such as the bone or soft tissue without any evidence of bone marrow or systemic involvement. Solitary plasmacytomas only comprise of 2–5% of all plasma cell dyscrasias, and extramedullary solitary plasmacytomas (ESPs) are rarer making up only 3–4% of all SPs, while other reports mention that incidence of solitary bone plasmacytomas (SBPs) is 40% higher than that of ESPs [1, 2]. The incidence of ESPs with anaplastic features such as our case is unknown as they are very rare.

ESPs most commonly occur in the 6^th^ to 8^th^ decade of life, and compared to bone plasmacytomas, there is a higher male predominance for an ESP of 3–4:1 [3, 4]. ESPs most commonly occur in the head and neck or the upper aerodigestive tract region. [5] Other sites of ESPs (extramedullary plasmacytomas) that are reported are the retroperitoneum, muscle, lungs, and GI tract. [6–8] The symptoms of ESPs depend upon the location of the tumors; in the head and neck area, they can range from pain, airway obstruction, anosmia, change of speech, dysphagia, or localized bleeding. The workup for an ESP should include serum and urine electrophoresis, serum immunofixation, free light chain assay, bone marrow biopsy, and a metastatic bone survey, either via X-rays or a PET scan.

The differential diagnosis for an ESP with anaplastic features includes plasmablastic lymphoma, ALK-positive large B-cell lymphoma, and extracavitary primary effusion lymphoma. These four neoplasms share a high-grade morphology with variable plasmacytoid morphologic features, express plasma cell markers, including CD38, CD138, and MUM-1, lack pan-B-cell markers such as CD19, CD20, and PAX-5, and show variable expression of CD45, CD79a, CD30, EMA, CD56, and heavy and light chains. Plasmablastic lymphoma characteristically occurs not only in immunocompromised patients, most commonly in HIV-positive patients, but also in older individuals and in association with iatrogenic immunosuppression as in posttransplant patients and patients with autoimmune diseases. It typically shows high proliferation with a Ki-67 close to a 100% and with increased apoptosis, sometimes imparting a starry-sky appearance. The majority of cases (60–75%) are positive for EBER by ISH, while CD56 expression is detected in about 25% of cases. ALK-positive large B-cell lymphoma is characterized, as the name implies, by ALK-1 positivity (mostly granular cytoplasmic staining pattern with few cases showing a cytoplasmic, nuclear, and nucleolar staining pattern), while extracavitary primary effusion lymphoma is characterized by coexpression of HHV8 and EBER by ISH. A high-grade neoplasm with plasmacytoid features which lacks HHV8, EBER by ISH, and ALK-1 expression in an immunocompetent patient is most likely to represent a plasma cell neoplasm, especially if a monoclonal protein, bone marrow involvement, or lytic bone lesions are present. It is of note that some plasma cell neoplasms (plasmacytoma or plasma cell myeloma) can express EBV, so a positive EBER by ISH should not be used in isolation, to establish the diagnosis of plasmablastic lymphoma, and a correlation with all clinical, laboratory, radiologic, morphologic, and immunohistochemical findings is required for definitive diagnosis; however, in some cases with overlapping features, a descriptive diagnosis of “plasmablastic neoplasm consistent with plasmablastic lymphoma or anaplastic plasmacytoma” may be acceptable, according to the 2016 WHO classification of tumor of hematopoietic and lymphoid tissues [9]. It is also of note that tumors resembling plasmablastic lymphoma can develop in a patient with a documented prior history of a plasma cell neoplasm, and these cases are best classified as plasmablastic transformation of a plasma cell neoplasm as opposed to a primary plasmablastic lymphoma.

Whether management should be more aggressive for this entity is unknown. Some case reports have shown that radiation or surgery alone will suffice; however, there are reports of this entity not responding to chemotherapy or radiation [6, 10, 11]. The location of the plasmacytoma may affect its response to different modalities of treatment. ESP still has a lower rate of converting into multiple myeloma when compared to solitary bone plasmacytoma (SBP), and a myeloma-free survival at10 yr for ESP was 71.2% while SBP was 0% [12]. Progression to multiple myeloma for ESP with anaplastic features is unknown.

The management of head and neck ESP includes resection of the tumor, radiation (RT) alone, or surgery followed by radiation. Head and neck ESP are radiosensitive with one case series of 18 patients treated with RT alone having local recurrence in only 1 patient [13]. Bachar et al. reviewed 68 cases and suggested that patients treated with radiation had an improved local free recurrence survival [2]. Sasaki et al. treated 67 patients with RT alone, and the 5-year and 10-year local control rates were 95% and 87%, respectively [14]. Another study revealed a 10-year local control of 88.9% with radiation alone [15]. The average dose of radiation should be at least 40–50 Gy given over 20–25 fractions. The role of chemotherapy in ESP is unknown. In the case report by Aluko et al., the patient had nasopharyngeal ESP similar to ours and was treated with 2 cycles of chemotherapy (cyclophosphamide, thalidomide, and dexamethasone) followed by radiation [1]. In the article by Bachar et al., 3 patients did receive concurrent chemoradiation as well with no difference in the outcomes when compared to RT alone [2]. Chemotherapy can be considered for high-grade tumors or large tumors as recurrence rates for higher grade ESP is high with RT alone. [16].

In our case, there was a strong consideration for adjuvant chemotherapy given the anaplastic features, but due to his comorbidities, we decided against it. We believe that RT alone should suffice for treatment of head and neck ESP; however, adjuvant chemotherapy should be considered in patients with anaplastic features. Surgery should only be performed for emergent issues or contraindications to RT.

## 4. Conclusions

Extramedullary plasmacytomas are very rare subtypes of plasma cell dyscrasias with anaplastic features being rarer. The head and neck, as well as the upper aerodigestive tract, are the most common sites of extramedullary plasmacytomas. Solitary bone plasmacytomas tend to have a higher rate of progressing to multiple myelomas when compared to extramedullary plasmacytomas. Extramedullary plasmacytomas of the head and neck region are radiosensitive, and in majority of the cases, radiation alone with a dose of approximately 40–50 Gy should be curative. However, ideal management is unknown. Extramedullary plasmacytomas with anaplastic features closely resemble plasmablastic lymphoma (PBL), and PBL needs to be ruled out because this tends to be more aggressive and usually requires systemic chemotherapy.

## Data Availability

The data can be obtained from the corresponding author upon request, and they will be given after discussing with the IRB; however, all the necessary information and images are in the main manuscript.
